# Serum Cytokines as Biomarkers in Heart Failure with Preserved Ejection Fraction and Sleep Apnea: A Prospective Cohort Study

**DOI:** 10.3390/life13030628

**Published:** 2023-02-23

**Authors:** Alexey Yakovlev, Alexander Teplyakov, Elena Grakova, Sergey Shilov, Natalia Yakovleva, Kristina Kopeva, Valery Shirinsky, Ivan Shirinsky

**Affiliations:** 1 Department of Therapy, Hematology and Transfusiology, Novosibirsk State Medical University, Krasny Prospect, 52, 630091 Novosibirsk, Russia; 2 Cardiology Department, Non-State Healthcare Institution “Road Clinical Hospital in Novosibirsk”, Russian Railways Joint Stock Company, Vladimirovsky Spusk, 630003 Novosibirsk, Russia; 3 Cardiology Research Institute, Tomsk National Research Medical Center (NRMC), Kievskaya Str., 111a, 634012 Tomsk, Russia; 4 Department of Myocardial Pathology, Cardiology Research Institute, Tomsk National Research Medical Center (NRMC), Kievskaya Str., 111a, 634012 Tomsk, Russia; 5 Department of Pathological Physiology and Clinical Pathophysiology, Novosibirsk State Medical University, Krasny Prospect, 52, 630091 Novosibirsk, Russia; 6 Department of Policlinic Therapy and General Medical Practice, Novosibirsk State Medical University, Krasny Prospect, 52, 630091 Novosibirsk, Russia; 7 Laboratory of Clinical Immunopharmacology, Federal State Budgetary Scientific Institution, Research Institute of Fundamental and Clinical Immunology, 6 Zalesskogo Str., 630099 Novosibirsk, Russia

**Keywords:** heart failure, cytokines, prognosis, sleep apnea, obstructive, biomarkers, comorbidity, phenotype, OSA, HFpEF, HF

## Abstract

Heart failure with preserved ejection fraction (HFpEF) and obstructive sleep apnea (OSA) frequently co-occur and this comorbidity represents a separate phenotype of HFpEF. While many research attempts are focused on biomarkers of HFpEF, currently, there is a lack of validated biomarkers of HFpEF and OSA. In this study, we aimed to evaluate prognostic significance of several serum cytokines in patients with HFpEF and OSA. The patients with HFpEF and OSA were recruited from the Sleep Apnea Center of Novosibirsk, Russian Federation and followed up for 12 months. The main analyzed outcomes were five-point major adverse cardiovascular events (MACE) and the 6-min walk test (6MWT). The analyzed cytokines were circulating IL-6, IL-10, and VEGF measured at baseline. We recruited 77 male patients with HFpEF and OSA, the data of 71 patients were available for analyses. Patients who developed MACE had four-fold elevated concentrations of serum IL-10. There was no association between baseline cytokine levels and longitudinal changes in 6MWT. Circulating IL-10 levels are positively associated with MACE in men with HFpEF and OSA and thus may be a potential prognostic biomarker in this subgroup of patients. These results should be confirmed in larger studies encompassing both males and females.

## 1. Introduction

Heart failure (HF) is a significant health problem with prevalence reaching 1–2% of the adult population in the Western world [1]. There are two major phenotypes of HF based on presence or absence of reduced left ventricular ejection fraction (LVEF)—HF with reduced ejection fraction (HFrEF) and HF with preserved ejection fraction (HFpEF). LVEF is a surrogate measurement of systolic function of the left ventricle of the heart and is frequently assessed using echocardiography [2]. HFpEF is defined as a “clinical syndrome in patients with current or prior symptoms of HF with a left ventricular ejection fraction (LVEF) ≥ 50 percent and evidence of cardiac dysfunction as a cause of symptoms”. Previously, HFpEF was termed “diastolic HF”, in contrast to HFrEF which was termed “systolic HF” [3]. HFrEF and HFpEF are pathogenetically distinct conditions and are associated with different biomarkers [4]. Currently, HFpEF is of particular concern forglobal healthcare [5] as its prevalence is rising [6] and there are still no effective treatment options for this type of HF [7].

HF is frequently accompanied with different comorbidities which can further impair prognosis of this condition [8]. One of the most important comorbidities in HF is sleep disordered breathing (SDB), which in turn can be central (central sleep apnea, CSA) or obstructive (obstructive sleep apnea, OSA) [9]. Both HFrEF and HFpEF can be linked to SDB. The predominant type of SDB in HFpEF is OSA, which occurs in up to 69–81% of these patients [10,11]. The relationships between HFpEF and OSA seem to be bidirectional. The hallmark of OSA is intermittent hopoxia. In spite of the short duration of hypoxia episodes, over the years, the cumulative burden of hypoxia-related changes becomes high. Hypoxia-induced tissue injury and lipid peroxidation cause systemic inflammation, triggering endothelial expression of adhesion molecules, which attracts monocytes, lowers endothelial production of nitric oxide, and raises endothelial production of reactive oxygen species. This in turn leads to local myocardial proinflammatory/fibrogenic signaling and finally, to myocardial fibrosis, which is the central mechanism of HFpEF [12]. On the other hand, HFpEF is associated with fluid retention [13]. Increased fluid accumulation in the neck results in narrowing of the pharynx and increasing its propensity to collapse during sleep. This represents a possible mechanism by which HFpEF can lead to increased risk of OSA [14].

There is a need for novel biomarkers for both HF [15] and OSA [16]. Due to high heterogeneity of HF and involvement of multiple factors in its pathogenesis, it can be assumed that each HF phenotype may be characterized by its own set of biomarkers. While a recent study for the first time evaluated serum biomarkers in HFrEF and CSA [17], there is a lack of research assessing biomarkers of HFpEF and OSA.

Given the role of cytokines in inflammation and angiogenesis in both HF [18] and SDB [19], we hypothesized that serum cytokines could be potential biomarkers in HFpEF and OSA. We chose three cytokines to evaluate as possible biomarkers. First, we used IL-6 as it is one of the major pro-inflammatory cytokines [20]. To characterize ongoing anti-inflammatory processes, we assessed serum IL-10 as a prototypical anti-inflammatory cytokine having a protective role against atherosclerosis [21]. For the assessment of angiogenesis, we chose vascular endothelial growth factor (VEGF), which is considered a pivotal regulator of angiogenesis [22]. We sought to evaluate an angiogenesis biomarker as recent research shows that coronary microvascular rarefaction (reduced myocardial capillary density) is a major contributor to diastolic dysfunction in HFpEF [23].

## 2. Materials and Methods

The study design was a prospective cohort study. The study protocol was approved by the Local Ethics Committee attached to the Clinical Hospital of Rossiyskie Zheleznye Dorogi, approval number 76. All patients provided written informed consent.

### 2.1. Patient Population

The patients were recruited from a population of male railroad workers from Novosibirsk Oblast attending an annual required medical checkup in the period from 2017 to 2019. People having three risk factors for OSA (BMI > 30, hypertension, snoring) were then referred to the Sleep Apnea Center for further evaluation. In order to improve efficiency of the screening, we did not use common screening tools like STOP-BANG, as in some studies, it lacked good performance in younger patients [24]. Thus, we used two objective risk factors of OSA (high BMI, verified hypertension) with one subjective factor, snoring, which was shown to be more associated with moderate/severe OSA than other risk factors [25].

The people attending the Sleep Apnea Center were invited to participate in the study. The patients were included in this study if, on baseline visit, they had confirmed HFpEF and OSA and fulfilled the following criteria:

### 2.2. Inclusion Criteria

(1) Symptoms of HF, New York Heart Association (NYHA) Functional Classification class I–II;
(2) Moderate to severe OSA (Apnea–Hypopnea Index (AHA)) > 15 in hour;
(3) Arterial hypertension;
(4) Abdominal obesity (waist circumference ≥ 92 cm, BMI ≥ 30;
(5) N-terminal (NT)-pro hormone BNP (NT-proBNP) > 125 pg/mL.

### 2.3. Exclusion Criteria

1. Reduced (≥50%) left ventricular ejection fraction;
2. Primary pulmonary hypertension;
3. History of pulmonary embolism with pulmonary hypertension ≥ 45 mm Hg;
4. Severe asthma or COPD;
5. Significant valvular abnormality;
6. Hypertrophic or dilated cardiomyopathy;
7. Coronary artery disease;
8. Persistent atrial fibrillation;
9. Thyroid disease, renal failure with creatinine clearance < 30 mL/m^2^;
10. Significant CSA (≥15 episodes of CSA in hour).

To diagnose OSA, all patients underwent polysomnography using the Somnolab2PSG diagnostic system (Weinemann, Germany). We used the Apnea Hypopnea Index (AHI) to assess the severity of OSA. To evaluate serum cytokines levels, we used the enzyme-linked immunosorbent assay (ELISA). Echocardiography was performed in all patients using standard protocol on the EPIQ device (Philips Ultrasound, Inc., Bothell, WA, USA).

### 2.4. Serum Cytokines

The ELISA analyses were performed using commercial ELISA kits (IL-6, IL-10, and VEGF ELISA-Best, Vector-Best, Novosibirsk, Russia). Based on the manufacturer’s instructions, the detection limits for IL-6, IL-10, and VEGF were 0.5 pg/mL, 2.5 pg/mL, and 10 pg/mL, respectively. Concentrations below these thresholds were considered non-detects.

The assessments were made on the baseline visit and on 12 months follow-up. On the baseline visit, we evaluated clinical and demographic parameters, polysomnography, echocardiography, 6-min walk test, and serum cytokine levels. On the follow-up visit, we re-performed all assessments except serum cytokines.

### 2.5. Outcomes

In this study, we assessed the following outcomes: five-point Major Adverse Cardiac Events (MACE) (primary endpoint) and six-minute walk test (6MWT) (secondary endpoint). The reason for choosing MACE as an outcome is that it is one of the most commonly used composite endpoints (CE) in both epidemiological studies and clinical trials on HF [26]. CE is defined as a “single measure of effect based on a combination of a variety of clinically relevant individual end points” [27]. The are many benefits of using CE as an outcome, including their clinical relevance, ease of use by all patients, capability of unbiased assessment, sensitivity, and low cost [27]. The choice of 6MWT was used because it is available, well-tolerated, and a highly reproducible test of functional capacity in HF patients [28].

### 2.6. Five-Point MACE

The criteria of five-point MACE were as follows [26]:

- Total death;
- Myocardial infarction;
- Stroke;
- Hospitalization because of HF;
- Revascularization, including percutaneous coronary intervention, and coronary artery bypass graft.

### 2.7. 6MWT

The 6MWT was performed according to the previous guidelines [29]. We advised patients to not engage in physical activities for 24 h and to not smoke or ingest alcohol for at least 3 h before the test. All 6MWT were performed outdoors along an 18-m corridor.

We instructed all patients to walk as fast as they could along the corridor. They were also informed to slow down their walk or to interrupt it if necessary. During the tests, we monitored the patients verbally by the Borg modified scale every 2 min. By the sixth minute, we requested each subject to stop and the number of runs and the remaining distance of the last run were summed. Before the test and during the first and sixth minute after finishing the test in the sitting position, we measured the blood pressure and H. Two tests were performed with a 30-min interval, and the average of the two 6MWD values was used to best approximate a representative true value.

### 2.8. Statistical Analysis

Serum cytokine values usually have a substantial proportion of non-detects. For a particular cytokine, we considered the concentrations below the reporting threshold as non-detectable and further treated them as left-censored. We performed Tobit regression, a recommended statistical approach for left-censored data [30] for each cytokine separately. This method allows adjustment for the effects of potential confounders such as age and BMI. Summary statistics and regression equations for the left-censored data were computed using maximum likelihood estimation (MLE) [31]. Using MLE allows data to be analyzed with up to 80% of censored values [32]. Using R package censReg version 0.5-26 [33], both the Student t test and censored regressions with and without potential confounders were performed to test the differences in cytokine levels between the patients with dichotomous outcomes (worsening of heart failure, hospital admission). In the case of continuous outcome (6MWT), we used censored regressions adjusted and non-adjusted for age and BMI. Due to the exploratory nature of this study, we did not perform sample size calculations.

## 3. Results

**Figure 1:**
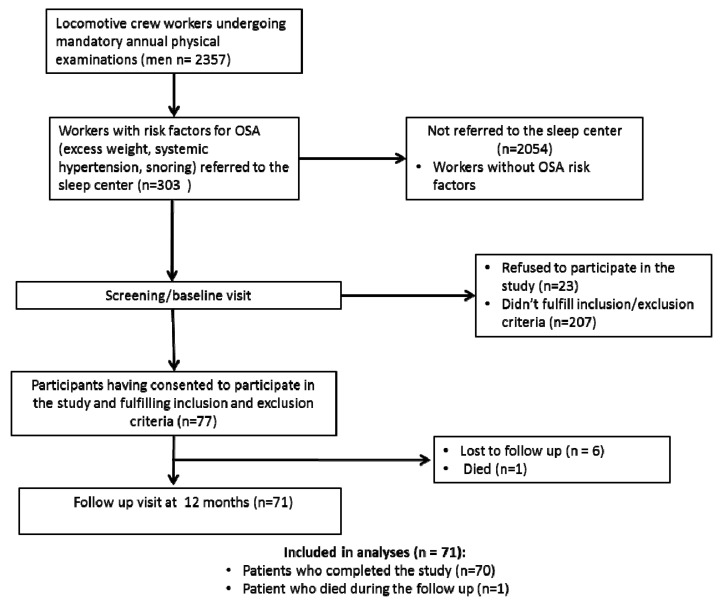
Study flow chart.

Figure 1 shows a flow chart of the participants. We enrolled 77 patients, 71 (92.2%) of them completed the follow-up, six (7.8%) were lost to follow-up, and one (1.4%) died. The patients who completed the study or died during the follow-up were included in the analyses.

The baseline characteristics of included patients are found in Table 1. All patients were middle aged men. Every fourth patient had concomitant COPD, every third patient was a smoker. All included patients had abdominal obesity and medication-controlled hypertension. Most of the patients had general (BMI > 30 kg/m^2^) and abdominal (waist circumference > 92 cm) obesity. All of the patients were diagnosed with NYHA grades I-II HF.

During the follow-up, 14 (19.17%) patients developed MACE. The incidence of individual components of MACE are presented in Table 2. As shown in the table, the hospitalization due to HF accounted for all cases of MACEs that occurred during the follow-up. In addition, one patient died due to worsening of HF, and one patient had a stroke.

Table 3 shows a comparison of baseline serum cytokine levels between patients with and without five-point MACE during the follow-up. In patients who developed MACE, the baseline concentrations of IL10 were four-fold higher; this difference was significant using the Student’s t test, and remained significant after adjustment for left censoring with Tobit regression modeling and after concomitant adjustment for age and BMI.

As shown in Table 4, there were no associations between baseline cytokine levels and changes in 6MWT over time.

## 4. Discussion

In this study, we found increased circulating IL-10 levels in patients with HFpEF and OSA who later developed MACE during the follow-up. There were no links between studied serum cytokines and physical function as measured by 6MWT.

To our knowledge, this was the first study evaluating serum cytokine biomarkers in a subset of patients with HFpEF and comorbid CSA.

Circulating cytokine levels have been assessed in many cross-sectional studies on OSA or HF. A recent meta-analysis showed increased circulating IL-6 levels in patients with OSA [34]. Reduced systemic levels of IL-10 were associated with the severity of OSA and insulin resistance [35] while VEGF levels were found to be frequently elevated in OSA [36]. In a large cohort of HF patients, elevated IL-6 levels were detected in more than 50% of patients [37]. In a recent study, patients with HFrEF exhibited a significant decrease in circulating VEGF [38]. IL-10 levels were also shown to be increased in HF patients [39].

Although many studies cross-sectionally assessed serum cytokines in HF and OS, there has been a lack of research prospectively evaluating prognostic value of IL-6, IL-10, and VEGF.

Our results conflict with the findings of the BIOSTAT-CHF study, showing IL-6 to be a predictor of worse CV outcomes [37]. The differences may be explained by the different study populations. Thus, the BIOSTAT-CHF study encompassed a more heterogeneous group of HF patients while our study focused on a subset of patients with HFpEF and comorbid CSA. It may therefore be hypothesized that different cytokines are involved in particular subtypes of HF.

Our findings are in line with the studies showing impaired CV outcomes in patients with elevated serum IL-10 [40,41,42]. These data have been difficult to interpret given the biology of IL-10, which is a prototypical anti-inflammatory cytokine [43] and theoretically should promote better cardiovascular outcomes [44]. The possible explanations for the positive associations between impaired CV outcomes and higher IL-10 levels are that the latter may exert unknown harmful action that could overcome any of its favorable anti-inflammatory effects. Alternatively, increased levels of this anti-inflammatory marker may represent a compensatory or counterregulatory mechanism. Any inflammation is accompanied by IL-10 production. The purpose of IL-10 in the setting of inflammation is to diminish excessive inflammation and to prevent unnecessary tissue damage [45]. Thus, higher IL-10 levels found in our study may represent a secondary increase of IL-10 production in response to higher inflammation [41].

From the first glance, the absence of a link between baseline cytokines and longitudinal changes in 6MWT contradicts our findings on elevated IL-10 in patients who had developed MACE. This discrepancy can be explained by suboptimal performance of 6MWT as an outcome in HF patients. Thus, a meta-analysis showed 6MWT improvement in only nine of 47 randomized controlled trials of pharmacological therapy in HF [46].

Our study has several important limitations: it was a single-center study, performed on a rather small sample of middle-aged men. These might restrict the generalizability of our findings.

The strengths of our study are that it had a longitudinal design and that we performed a statistical analyses accounting for left censoring inherent to immunological data.

This study represents the first screening step in evaluating IL-10 as a candidate predictor biomarker of adverse CV outcomes in patients with HFpEF with OSA. The future studies on larger populations will need to confirm these results. If IL-10 is confirmed to be significantly elevated in patients with increased incidence of negative CV outcomes, further research will have to evaluate the effectiveness of serum IL-10 as a predictive biomarker. Determining clinical cutoffs using receiver operating characteristics analysis will be necessary to allow using serum cytokines as biomarkers in clinical practice.

An “ideal” predictive biomarker should be noninvasive, inexpensive, and effective [47]. The first two requirements are probably met for serum cytokines as their measurement requires a draw of just a small amount of venous blood and quantification using ELISA is relatively inexpensive. The effectiveness of the measurement of serum cytokines for the prediction of clinical outcomes is more questionable. Using serum cytokines as a biomarker in clinical settings is challenging due to several reasons. Serum cytokine concentrations might be affected by comorbid diseases. Blood cytokines have a short half-life, their blood levels are relatively low [48]. In addition, there is marked variability in serum cytokine concentrations, including between-day [49] and diurnal [50] variability.

In spite of these challenges, there is still a hope that some cytokines can be used as effective biomarkers for some diseases in the future. The reason for this hope is that there are at least several probable ways to overcome the said difficulties: using a combination of several cytokines to build more accurate predictive models, establishing valid reference cytokine concentrations on large populations of healthy people, and a rigorous standardization of the measurement techniques [51,52].

In conclusion, higher serum concentrations of IL10 in men with HFpEF and CSA are associated with MACE during the follow-up. These findings need to be replicated on a more general population with a larger sample size.

## Figures and Tables

**Table 1:** Baseline characteristics of patients.

*n*	71
Age, years	46.5 (10.7)
Male	71 (100)
COPD	18 (25.35)
Smoking	27 (38.03)
Dyslipidemia	32 (43.8)
Diabetes mellitus type 2	12 (16.4)
6MWT, m	494.21 (100.8)
BMI, kg/m^2^	34.26 (5.72)
NYHA class: I II III IV	33 (46.48) 28 (38.36) 0 0
Hypertension	71 (100)
Paroxismal atrial fibrillation	13 (17.8)
Cardiovascular medication profile	
ACE inhibitors	43 (58.9)
Angiotensin 2 receptor antagonists	35 (47.95)
Beta blockers	45 (61.64)
Diuretics	35 (47.95)
Calcium channel blockers	29 (39.73)

Data are presented as the mean (SD) or *n* (%). HF: heart failure, NYHA: New York Heart Association (NYHA) Functional Classification, VPB: ventricular premature beats, BP: blood pressure, ACE: Angiotensin-converting enzyme, SD: standard deviation.

**Table 2:** Components of major adverse cardiovascular events during the follow-up (*n* = 14).

Cardiovascular Event	*n*, %
Total death	1 (7.1)
Myocardial infarction	0 (0)
Stroke	1 (7.1)
Hospitalization because of HF	14 (100)
Revascularization, including percutaneous coronary intervention, and coronary artery bypass graft.	0 (0)

HF: heart failure.

**Table 3:** Association between baseline serum cytokines and five-point major adverse cardiac events during the follow-up.

Cytokine	Patients without MACE (*n* = 57)	Patients with MACE (*n* = 14)	*p* Value ^a^	*p* Value ^b^	*p* Value ^c^
IL6, pg/mL	5.29 (11.70)	6.23 (12.22)	0.790	0.493	0.674
VEGF, pg/mL	339.34 (274.51)	405.02 (178.59)	0.398	0.371	0.5742
IL10, pg/mL	6.85 (8.80)	26.01 (55.88)	0.014	0.0102	0.00574

Cytokine concentrations are presented as mean (SD). ^a^ Student’s *t* test, ^b^ Tobit regression taking censoring into account, ^c^ Tobit regression taking censoring into account and adjusting for age and BMI. MACE: Major adverse cardiac events, IL: interleukin, VEGF: Vascular endothelial growth factor, SD: standard deviation.

**Table 4:** Associations between baseline serum cytokines and changes in 6MWT after 1 year of follow-up.

	Linear Regression		Non-Adjusted Tobit Regression		Tobit Regression Adjusted for Age and BMI	
	β-Coefficient (95 CI)	*p* Value	β-Coefficient (95 CI)	*p* Value	β-Coefficient (95 CI)	*p* Value
IL-6	−0.01 (−0.1–0)	0.6	−0.02 (−0.1–0)	0.34	0.01 (−0.1–0)	0.63
VEGF	−0.01 (−1.1–1.0)	0.86	0 (−1.2–1.0)	0.9	0.01 (−1.1–1.1)	0.19
IL-10	−0.02 (−0.1–0.1)	0.67	−0.02 (−0.1–0.1)	0.65	−0.13 (−0.1–0.1)	0.59

IL: interleukin, VEGF: Vascular endothelial growth factor, CI: confidence interval.

## Data Availability

The data presented in this study are available on request from the corresponding author. The data are not publicly available due to privacy and ethical restrictions.

## References

[1] Mosterd A., Hoes A.W. (2007). Clinical epidemiology of heart failure. Heart.

[2] Solomon S.D., Anavekar N., Skali H., McMurray J.J., Swedberg K., Yusuf S., Granger C.B., Michelson E.L., Wang D., Pocock S. (2005). Influence of ejection fraction on cardiovascular outcomes in a broad spectrum of heart failure patients. Circulation.

[3] Borlaug B. (2022). Heart failure with preserved ejection fraction: Clinical manifestations and diagnosis. UpToDate.

[4] Tromp J., Khan M.A., Klip I.T., Meyer S., de Boer R.A., Jaarsma T., Hillege H., van Veldhuisen D.J., van der Meer P., Voors A.A. (2017). Biomarker Profiles in Heart Failure Patients With Preserved and Reduced Ejection Fraction. J. Am. Heart Assoc..

[5] Naing P., Forrester D., Kangaharan N., Muthumala A., Mon Myint S., Playford D. (2019). Heart failure with preserved ejection fraction: A growing global epidemic. Aust. J. Gen. Pract..

[6] Owan T.E., Hodge D.O., Herges R.M., Jacobsen S.J., Roger V.L., Redfield M.M. (2006). Trends in prevalence and outcome of heart failure with preserved ejection fraction. N. Engl. J. Med..

[7] Roh J., Houstis N., Rosenzweig A. (2017). Why Don’t We Have Proven Treatments for HFpEF?. Circ. Res..

[8] Kheirbek R.E., Alemi F., Fletcher R. (2015). Heart failure prognosis: Comorbidities matter. J. Palliat. Med..

[9] Cowie M.R., Gallagher A.M. (2017). Sleep Disordered Breathing and Heart Failure: What Does the Future Hold?. JACC Heart Fail..

[10] Bitter T., Faber L., Hering D., Langer C., Horstkotte D., Oldenburg O. (2009). Sleep-disordered breathing in heart failure with normal left ventricular ejection fraction. Eur. J. Heart Fail..

[11] Herrscher T.E., Akre H., Overland B., Sandvik L., Westheim A.S. (2011). High prevalence of sleep apnea in heart failure outpatients: Even in patients with preserved systolic function. J. Card. Fail..

[12] Paulus W.J., Zile M.R. (2021). From Systemic Inflammation to Myocardial Fibrosis: The Heart Failure With Preserved Ejection Fraction Paradigm Revisited. Circ. Res..

[13] Fudim M., Ashur N., Jones A.D., Ambrosy A.P., Bart B.A., Butler J., Chen H.H., Greene S.J., Reddy Y., Redfield M.M. (2021). Implications of peripheral oedema in heart failure with preserved ejection fraction: A heart failure network analysis. ESC Heart Fail..

[14] Levy P., Naughton M.T., Tamisier R., Cowie M.R., Bradley T.D. (2022). Sleep apnoea and heart failure. Eur. Respir. J..

[15] Ibrahim N.E., Januzzi J.L. (2018). Established and Emerging Roles of Biomarkers in Heart Failure. Circ. Res..

[16] Lorenzi-Fillho G., Drager L.F. (2010). Sleep apnea: Why should we look for cardiac biomarkers?. J. Clin. Sleep Med..

[17] Ferreira J.P., Duarte K., Woehrle H., Cowie M.R., Wegscheider K., Angermann C., d’Ortho M.P., Erdmann E., Levy P., Simonds A.K. (2020). Biomarkers in patients with heart failure and central sleep apnoea: Findings from the SERVE-HF trial. ESC Heart Fail..

[18] Suthahar N., Meijers W.C., Sillje H.H.W., de Boer R.A. (2017). From Inflammation to Fibrosis-Molecular and Cellular Mechanisms of Myocardial Tissue Remodelling and Perspectives on Differential Treatment Opportunities. Curr. Heart Fail. Rep..

[19] Kheirandish-Gozal L., Gozal D. (2019). Obstructive Sleep Apnea and Inflammation: Proof of Concept Based on Two Illustrative Cytokines. Int. J. Mol. Sci..

[20] Fernando M.R., Reyes J.L., Iannuzzi J., Leung G., McKay D.M. (2014). The pro-inflammatory cytokine, interleukin-6, enhances the polarization of alternatively activated macrophages. PLoS ONE.

[21] Han X., Boisvert W.A. (2015). Interleukin-10 protects against atherosclerosis by modulating multiple atherogenic macrophage function. Thromb. Haemost..

[22] Dabravolski S.A., Khotina V.A., Omelchenko A.V., Kalmykov V.A., Orekhov A.N. (2022). The Role of the VEGF Family in Atherosclerosis Development and Its Potential as Treatment Targets. Int. J. Mol. Sci..

[23] Zeng H., Chen J.X. (2019). Microvascular Rarefaction and Heart Failure With Preserved Ejection Fraction. Front. Cardiovasc. Med..

[24] Lyons R., Barbir L.A., Owens R., Colvonen P.J. (2022). STOP-BANG screener vs objective obstructive sleep apnea testing among younger veterans with PTSD and insomnia: STOP-BANG does not sufficiently detect risk. J. Clin. Sleep Med..

[25] Borsini E., Ernst G., Salvado A., Bosio M., Chertcoff J., Nogueira F., Nigro C. (2015). Utility of the STOP-BANG components to identify sleep apnea using home respiratory polygraphy. Sleep. Breath.

[26] Bosco E., Hsueh L., McConeghy K.W., Gravenstein S., Saade E. (2021). Major adverse cardiovascular event definitions used in observational analysis of administrative databases: A systematic review. BMC Med. Res. Methodol..

[27] Gomez G., Gomez-Mateu M., Dafni U. (2014). Informed choice of composite end points in cardiovascular trials. Circ. Cardiovasc. Qual. Outcomes.

[28] Giannitsi S., Bougiakli M., Bechlioulis A., Kotsia A., Michalis L.K., Naka K.K. (2019). 6-minute walking test: A useful tool in the management of heart failure patients. Ther. Adv. Cardiovasc. Dis..

[29] (2002). ATS statement: Guidelines for the six-minute walk test. Am. J. Respir. Crit. Care Med..

[30] Ballenberger N., Lluis A., von Mutius E., Illi S., Schaub B. (2012). Novel statistical approaches for non-normal censored immunological data: Analysis of cytokine and gene expression data. PLoS ONE.

[31] Helsel D.R. (2005). Nondetects and Data Analysis: Statistics for Censored Environmental Data.

[32] Huynh T., Ramachandran G., Banerjee S., Monteiro J., Stenzel M., Sandler D.P., Engel L.S., Kwok R.K., Blair A., Stewart P.A. (2014). Comparison of methods for analyzing left-censored occupational exposure data. Ann. Occup. Hyg..

[33] Henningsen A. censReg: Censored Regression (Tobit) Models. https://CRAN.R-project.org/package=censReg.

[34] Imani M.M., Sadeghi M., Khazaie H., Emami M., Sadeghi Bahmani D., Brand S. (2020). Evaluation of Serum and Plasma Interleukin-6 Levels in Obstructive Sleep Apnea Syndrome: A Meta-Analysis and Meta-Regression. Front. Immunol..

[35] Leon-Cabrera S., Arana-Lechuga Y., Esqueda-Leon E., Teran-Perez G., Gonzalez-Chavez A., Escobedo G., Velazquez Moctezuma J. (2015). Reduced systemic levels of IL-10 are associated with the severity of obstructive sleep apnea and insulin resistance in morbidly obese humans. Mediat. Inflamm..

[36] Gozal D., Lipton A.J., Jones K.L. (2002). Circulating vascular endothelial growth factor levels in patients with obstructive sleep apnea. Sleep.

[37] Markousis-Mavrogenis G., Tromp J., Ouwerkerk W., Devalaraja M., Anker S.D., Cleland J.G., Dickstein K., Filippatos G.S., van der Harst P., Lang C.C. (2019). The clinical significance of interleukin-6 in heart failure: Results from the BIOSTAT-CHF study. Eur. J. Heart Fail..

[38] Chaar D., Dumont B., Vulesevic B., Neagoe P.E., Rakel A., Sirois M.G., White M. (2021). Neutrophils pro-inflammatory and anti-inflammatory cytokine release in patients with heart failure and reduced ejection fraction. ESC Heart Fail..

[39] Loppnow H., Werdan K., Werner C. (2002). The enhanced plasma levels of soluble tumor necrosis factor receptors (sTNF-R1; sTNF-R2) and interleukin-10 (IL-10) in patients suffering from chronic heart failure are reversed in patients treated with beta-adrenoceptor antagonist. Auton. Autacoid. Pharmacol..

[40] Yilmaz M.I., Solak Y., Saglam M., Cayci T., Acikel C., Unal H.U., Eyileten T., Oguz Y., Sari S., Carrero J.J. (2014). The relationship between IL-10 levels and cardiovascular events in patients with CKD. Clin. J. Am. Soc. Nephrol..

[41] Cavusoglu E., Marmur J.D., Hojjati M.R., Chopra V., Butala M., Subnani R., Huda M.S., Yanamadala S., Ruwende C., Eng C. (2011). Plasma interleukin-10 levels and adverse outcomes in acute coronary syndrome. Am. J. Med..

[42] Amir O., Rogowski O., David M., Lahat N., Wolff R., Lewis B.S. (2010). Circulating interleukin-10: Association with higher mortality in systolic heart failure patients with elevated tumor necrosis factor-alpha. Isr. Med. Assoc. J..

[43] Moore K.W., de Waal Malefyt R., Coffman R.L., O’Garra A. (2001). Interleukin-10 and the interleukin-10 receptor. Annu. Rev. Immunol..

[44] Tedgui A., Mallat Z. (2006). Cytokines in atherosclerosis: Pathogenic and regulatory pathways. Physiol. Rev..

[45] Couper K.N., Blount D.G., Riley E.M. (2008). IL-10: The master regulator of immunity to infection. J. Immunol..

[46] Olsson L.G., Swedberg K., Clark A.L., Witte K.K., Cleland J.G. (2005). Six minute corridor walk test as an outcome measure for the assessment of treatment in randomized, blinded intervention trials of chronic heart failure: A systematic review. Eur. Heart J..

[47] Yang C., Luo G., Cheng H., Lu Y., Jin K., Wang Z., Liu C., Yu X. (2018). Potential biomarkers to evaluate therapeutic response in advanced pancreatic cancer. Transl. Cancer Res..

[48] Radonjic-Hoesli S., Pavlov N., Simon H.U., Simon D. (2022). Are blood cytokines reliable biomarkers of allergic disease diagnosis and treatment responses?. J. Allergy Clin. Immunol..

[49] Rose G.L., Farley M.J., Flemming N.B., Skinner T.L., Schaumberg M.A. (2022). Between-day reliability of cytokines and adipokines for application in research and practice. Front. Physiol.

[50] Altara R., Manca M., Hermans K.C., Daskalopoulos E.P., Brunner-La Rocca H.P., Hermans R.J., Struijker-Boudier H.A., Blankesteijn M.W. (2015). Diurnal rhythms of serum and plasma cytokine profiles in healthy elderly individuals assessed using membrane based multiplexed immunoassay. J. Transl. Med..

[51] Monastero R.N., Pentyala S. (2017). Cytokines as Biomarkers and Their Respective Clinical Cutoff Levels. Int. J. Inflamm..

[52] Liu C., Chu D., Kalantar-Zadeh K., George J., Young H.A., Liu G. (2021). Cytokines: From Clinical Significance to Quantification. Adv. Sci..

